# Antimicrobial and Selected *In Vitro* Enzyme Inhibitory Effects of Leaf Extracts, Flavonols and Indole Alkaloids Isolated from *Croton menyharthii*

**DOI:** 10.3390/molecules181012633

**Published:** 2013-10-11

**Authors:** Mutalib A. Aderogba, Ashwell R. Ndhlala, Kannan R. R. Rengasamy, Johannes Van Staden

**Affiliations:** 1 Research Centre for Plant Growth and Development, School of Life Sciences, University of KwaZulu-Natal Pietermaritzburg, Private Bag X01, Scottsville 3209, South Africa; E-Mails: Aderogba@ukzn.ac.za (M.A.A.); ndhlala@ukzn.ac.za (A.R.N.); rengasamy@ukzn.ac.za (K.R.R.R.); 2 Department of Chemistry, Obafemi Awolowo University, Ile-Ife 220005, Nigeria

**Keywords:** *Croton menyharthii* Pax, flavonols, alkaloid, COX-2, antimicrobial, alpha-glucosidase, mutagenicity

## Abstract

*Croton* species are used in folk medicine in the management of infections, inflammation and oxidative stress-related diseases. In order to isolate, characterize and evaluate the bioactive constituents of *Croton menyharthii* Pax leaf extracts, repeated column fractionation of the ethyl acetate fraction from a 20% aqueous methanol crude extract afforded three flavonols identified by NMR (1D and 2D) spectroscopic methods as myricetrin-3-*O*-rhamnoside (myricetrin, **1**), quercetin-3-*O*-rhamnoside (**2**) and quercetin (**3**) along with an indole alkaloid, (E)-*N*-(4-hydroxycinnamoyl)-5-hydroxytryptamine, [*trans*-N-(*p*-coumaroyl) serotonin, **4**]. All the compounds are reported from the leaf extract of this plant for the first time. The crude extracts, four solvent fractions (hexane, DCM, ethyl acetate and butanol) and isolated compounds obtained from the leaves were evaluated for their inhibitory effects on selected bacteria, a fungus (*Candida albicans*), cyclooxygenase (COX-2), α-glucosidase and acetylcholinesterase (AChE). Amongst the compounds, quercetin (**3**) was the most active against *Bacillus subtilis* and *Candida albicans* while myricetrin-3-*O*-rhamnoside (**1**) and *trans*-N-(*p*-coumaroyl) serotonin (**4**) were the most active compounds against *Escherichia coli*, *Klebsiella pneumonia* and *Staphylococcus aureus*. The inhibitory activity of myricetrin-3-*O*-rhamnoside (**1**) against COX-2 was insignificant while that of the other three compounds **2**–**4** was low. The AChE inhibitory activity of the alkaloid, *trans*-*N*-(*p*-coumaroyl) serotonin was high, with a percentage inhibitory activity of 72.6% and an IC_50_ value of 15.0 µg/mL. The rest of the compounds only had moderate activity. *Croton menyharthii* leaf extracts and isolated compounds inhibit α-glucosidase at very low IC_50_ values compared to the synthetic drug acarbose. Structure activity relationship of the isolated flavonols **1**–**3** is briefly outlined. Compounds **1**–**4** and the leaf extracts exhibited a broad spectrum of activities. This validates the ethnomedicinal use of the plant in folk medicine.

## 1. Introduction

The genus *Croton* belongs to the family Euphorbiaceae. Many species are used in traditional medicine in the management of diabetes, hypertension, inflammation, infections, cancer and fever. In pharmacological assays *Croton* extracts and constituents have exhibited various biological activities, including hypoglycaemic, anti-cancer, anti-hypertensive, anti-inflammatory, antimalarial and antimicrobial effects [1]. The observed activities provide a rationale for the ethnomedicinal use of *Croton* species in folk medicine. *Croton menyharthii* pax parts (roots, root bark and leaves) are used in management of dysmenorrhoea, intestinal obstruction, hepatitis and ascites [2], but previous pharmacological studies on the leaf extracts did not validate ethnomedicinal use of the plant, as no significant antibacterial and antifungal activities were noted for the aqueous extracts [3]. However, a mosquito repellent effect of the plant was recently documented [4]. To the best of our knowledge there are only scanty reports on the pharmacological effects and none on the isolation and identification of the active constituents of *C. menyharthii* roots, root bark, stem bark and leaf extracts. The purpose of this study was to investigate the extracts and constituents of *C. menyharthii* leaves for potential antimicrobial, inflammation and α-glucosidase inhibitory activities. These could be helpful in ameliorating some of these conditions and also provide a rationale for the ethnomedicinal use of the plant. Safety of the extracts and isolated compounds from the plant was investigated by determining their mutagenicity.

## 2. Results and Discussion

**Figure 1:**
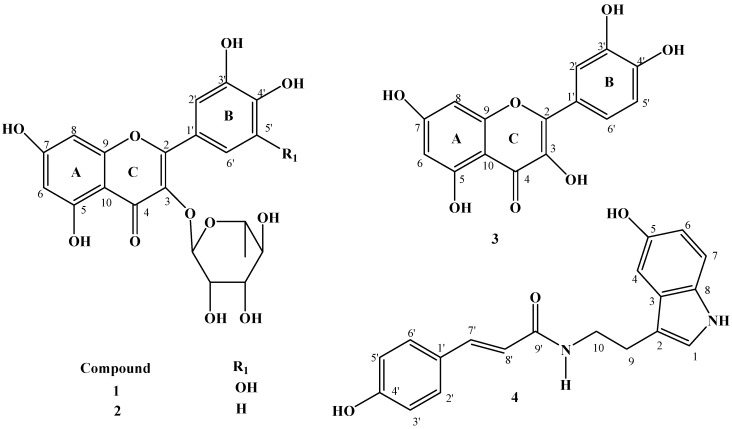
Structures of isolated compounds from *C. menyharthii* leaf extracts.

*Croton* species are used in folk medicine in the management of infections, inflammation and oxidative stress-related diseases such as diabetes [1]. In our efforts to find phytochemical agents that could be helpful in management of some of these conditions, we have investigated *Croton menyharthii* leaf extracts for its bioactive constituents. Three flavonols **1**–**3** and an indole alkaloid **4** were isolated from the leaf extracts of *C. menyharthii.* Structure elucidation of the isolated compounds was carried out using-NMR (1D and 2D) spectroscopic techniques and they were identified as myricetrin-3-*O*-rhamnoside (myricetrin, **1**), quercetin-3-*O*-rhamnoside (**2**) and quercetin (**3**) along with the indole alkaloid (*E*)-*N*-(4-hydroxycinnamoyl)-5-hydroxytryptamine, [*trans*-*N*-(*p*-coumaroyl)serotonin, **4**]. All the compounds are reported from the leaf extract of this plant for the first time. Their structures are presented in Figure 1.

### 2.1. Antimicrobial Bioassay

The MIC values for the methanol crude leaf extract, fractions and isolated compounds of *Croton menyharthii* are presented in Table 1. The plant extracts with high antibacterial activity (<1 mg/mL) [5], are highlighted in bold. Quercetin was the most effective compound against *Bacillus subtilis* and *Candida albicans* while myricetrin-3-*O*-rhamnoside (**1**) and *trans*-*N*-(*p*-coumaroyl) serotonin (**4**) were the most active compounds against *Escherichia coli*, *Klebsiella pneumonia* and *Staphylococcus aureus*. The varying structures of flavonoids dramatically affect their behavioural activities both *in vitro* and *in vivo*, such as absorption, metabolism and excretion [6]. Myricetrin-3-*O*-rhamnoside (**1**) and quercetin-3-*O*-rhamnoside (**2**), both naturally occurring flavonols, differ from quercetin only by the addition of a sugar moiety (rhamnose) at 3-OH and additional hydroxyl at the 5'-OH of the phenyl moiety (ring B) for myricetrin-3-*O*-rhamnoside (**1**). From the observed antimicrobial activity, the addition of the sugar moiety likely reduces the activity of the two compounds making their performance to be far less than that of quercetin against *Bacillus subtilis* and *Candida albicans*. The ethyl acetate fraction exhibited the highest antimicrobial activity against *Bacillus subtilis*, *Escherichia coli*, *Klebsiella pneumonia* and *Candida albicans*.

**Table 1:** Antimicrobial properties (MIC-mg/mL) of the isolated compounds, crude extracts and fractions from the methanol extracts of *Croton menyharthii* (n = 3).

Sample	Antimicrobial MIC (mg/mL)				
	*B.s.*	*E.c.*	*K.p.*	*S.a.*	*C.a.*
Myricetrin-3-*O*-rhamnoside (**1**)	**0.25**	**0.25**	**0.25**	**0.25**	**0.25**
Quercetin-3-*O*-rhamnoside (**2**)	**0.13**	>0.25	>0.25	>0.25	**0.25**
Quercetin (**3**)	**0.03**	>0.25	0.25	>0.25	**0.02**
*Trans*-*N*-(*p*-coumaroyl) serotonin (**4**)	**0.25**	**0.25**	**0.25**	**0.25**	**0.13**
*Croton menyharthii* crude	3.13	1.56	3.13	1.56	6.25
*Croton menyharthii* hexane	**0.78**	**0.78**	1.56	**0.78**	**0.78**
*Croton menyharthii* DCM	**0.39**	**0.78**	1.56	**0.78**	**0.78**
*Croton menyharthii* ethyl acetate	**0.39**	**0.39**	**0.39**	**0.78**	**0.39**
*Croton menyharthii* butanol	1.56	3.13	1.56	3.13	3.13
Neomycin	1.6 × 10^−3^	0.8 × 10^−3^	0.8 × 10^−3^	1.6 × 10^−3^	
Amphotericin B					9.77 × 10^−3^

B.s.: *Bacillus subtilis*; E.c.: *Escherichia coli*; K.p.: *Klebsiella pneumonia*; S.a.: *Staphylococcus aureus*, C.a.: *Candida albicans.* Samples with MIC values written in bold font are considered to be very active (MIC < 1 mg/mL).

### 2.2. Enzyme Inhibition Bioassay Results

**Figure 2:**
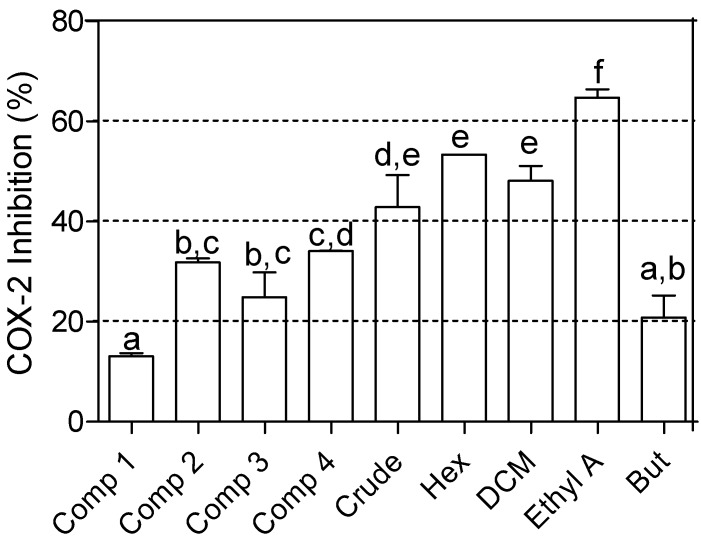
The percentage inhibition of COX-2 by the isolated compounds, crude extract and fractions of *Croton menyharthii*. Comp (at concentrations of 25 µg/mL) **1**, [myricetrin-3-*O*-rhamnoside]; **2**, [quercetin-3-*O*-rhamnoside]; **3**, [quercetin]; **4**, [*trans*-*N*-(*p*-coumaroyl) serotonin]; Crude, crude methanol extract; Hex, hexane; DCM, dichloromethane; Ethyl A, ethyl acetate and But, butanol (Crude extract and fractions were at concentrations of 250 µg/mL). The percentage inhibition by indomethacin (positive control) at a concentration of 20 µg/mL was 84.10 ± 0.17. Bars with similar letters are not significantly different at *p* ≤ 0.05.

The inhibitory effects on COX-2 enzyme by the methanol crude extract, fractions and isolated compounds of *Croton menyherthaii* leaves are presented in Figure 2.

Four levels of activity are defined in the COX assay with activity below 20% being considered insignificant, 20%–40% low, 40%–70% moderate and 70%–100% high [7]. The inhibitory activity of myricetrin-3-*O*-rhamnoside (**1**) was insignificant while that of the other three compounds **2**–**4** was low. The crude extract exhibited a moderate activity, while that of the butanol fraction was low. The DCM, hexane and ethyl acetate fractions had moderate activity, with the ethyl acetate being the highest.

The results of the AChE inhibitory activity (% and IC_50_) are presented in Table 2. Four levels of activity were defined in the assay with activity below 20% being considered insignificant, 20%–40% low, 40%–70% moderate and 70%–100% high. The AChE inhibitory activity of *trans*-*N*-(*p*-coumaroyl) serotonin was high, with a percentage inhibitory activity of 72.6% and an IC_50_ value of 15.0 µg/mL. The rest of the compounds had only moderate activity. The crude extract exhibited a moderate AChE inhibitory activity, also confirmed by a higher IC_50_ value. Amongst the fractions, the ethyl acetate fraction exhibited high AChE inhibitory activity while the rest showed moderate activity. The complexation of flavonoids and other phenolic compounds with proteins has been extensively investigated and are now known to structurally interact with enzymes to reduce protein digestibility, drug metabolism, reduce progression of diseases which involve enzymes in their advancement for example COX and AChE involved in the inflammation processes and Alzheimer’s disease (AD) respectively. On the other hand, information on interaction between phenolics and polysaccharides or glycosylated phenolics are scarcely reported [6]. The OH groups on the flavonoids and alkaloids are essential for bioactivity, and the change of the position or number of such groups affects biological potency. The plant-derived flavonoid quercetin is a broad-spectrum protein inhibitor [6].

**Table 2:** AChE and α-glucosidase inhibitory activity (IC_50_ µg/mL) of the crude extract, fractions and four compounds isolated from *Croton menyharthii* leaf methanol extract.

Sample name	AChE inhibitory activity		α-Glucosidase inhibitory activity	
	Percentage inhibition *	IC_50_ (µg/mL)	Percentage inhibition *	IC_50_ (µg/mL)
Myricetrin-3-*O*-rhamnoside (myricetrin) (**1**)	54.3 ± 1.1 b	65.0 ± 7.7 d	89.1 ± 5.3 e	79.0 ± 4.3 a,b
Quercetin-3-*O*-rhamnoside (**2**)	53.3 ± 1.0 b,c	60.7 ±7.9 d	47.0 ± 1.7 a	122.7 ± 1.6 a,b
Quercetin (**3**)	56.6 ± 0.3 c	41.6 ± 6.0 c	67.1 ± 4.2 b	30.9 ± 8.4 a,b
*Trans*-*N*-(*p*-coumaroyl) serotonin (**4**)	72.6 ± 4.9 f	15.0 ± 0.8 b	97.3 ± 3.2 f	5.3 ± 0.3 a
*Croton menyharthii* crude	48.2 ± 1.8 a	988.4 ± 12.6 i	98.3 ± 1.4 f	43.7 ± 2.2 a,b
*Croton menyharthii* hexane	63.4 ± 3.4 e	658.3 ± 8.2 g	95.1 ± 1.5 f	55.5 ± 10.5 a,b
*Croton menyharthii* DCM	60.5 ± 2.2 d	257.5 ± 9.1 f	95.6 ± 4.7 f	47.5 ± 0.4 b,c
*Croton menyharthii* ethyl acetate	81.4 ± 1.6 g	105.0 ± 2.0 e	91.1 ± 2.2 e	90.3 ± 0.9 a,b
*Croton menyharthii* butanol	52.7 ± 0.8 b	768.6 ± 5.6 h	72.1 ± 3.5 c	366.3 ± 107.1 c
Galanthamine	88.3 ± 2.0 h	0.3 ± 0.1 a		
Acarbose			83.6 ± 2.6 d	103.3 ± 9.3 a,b

* Percentage inhibition for AChE and α-glucosidase inhibitory activity was at a concentration of 1,000 µg/mL for crude extract and fractions, 300 µg/mL for pure compounds and 20 µg/mL for galanthamine (positive control) and 250 µg/mL for acarbose (positive control). Results are expressed as means ± standard errors of two independent experiments, each experiment in duplicate. Values in a column with similar letters are not significantly different at *p* ≤ 0.05.

α-Glucosidase plays a major role in the management of type 2 diabetes by delaying carbohydrate metabolism. Many species of the *Croton* family, including *C. Lobatus*, Linn [8], *Croton cajucara* Benth [9], *C. zambesicus* Müll.Arg [10] and *C. cuneatus* Klotz. [11] are widely used in traditional medicine to treat diabetes mellitus. So far there are no reports on the antidiabetic effects of *C. menyharthii.* The inhibitory effect of the crude *C. menyharthii* extract and different solvent fractions against α-glucosidase was evaluated to access the antidiabetic potential of this species. Crude extract and all the solvent fractions exhibited strong inhibitory activity against α-glucosidase, with IC_50_ values ranging from 43.7 to 366.3 µg/mL when compared to the positive control acarbose. The butanol fraction showed less activity (Table 2). The results are presented in Table 2. All the isolated compounds were more active than the positive control acarbose except compound **2** which showed less activity than acarbose. The indole alkaloid, *trans*-*N*-(*p*-coumaroyl) serotonin (**4**) exhibited promising inhibitory effect at the lowest IC_50_ value of 5.3 µg/mL. It is well known that alkaloids are prone to inhibit enzymes and it has been proved that various alkaloids isolated from plant sources exhibited very strong α-glucosidase inhibitory effects compared to synthetic drugs used for the treatment of diabetes [12,13]. The hydroxyl on the 3-position of the flavonoid plays an important role in the inhibitory activity against α-glucosidase. The glycosylation of the 3-OH of the flavonols has been demonstrated unfavourable to the inhibitory activity [14]. This is evidenced with quercetin, in that while possessing the 3-OH, showed higher inhibitory activity than the 3-*O*-glycoside compounds: myricetrin-3-*O*-rhamnoside (**1**) and quercetin-3-*O*-rhamnoside (**2**). Additional 5'-OH on ring B of myricetrin-3-*O*-rhamnoside (**1**) could account for its higher activity than that of quercetin-3-*O*-rhamnoside (**2**). Flavonoids are reported as potential antidiabetic agents due to their multiple actions that are both hypoglyceamic and antihyperglyceamic [15].

## 3. Experimental

### 3.1. General

All thin layer chromatography analyses were performed at room temperature using pre-coated plates (MERCK, silica gel 60 F_254_ 0.2 thickness). Detection of spots was done by viewing under UV light (254 and 366 nm). Open column chromatography was carried out using silica gel (230-400 mesh) and Sephadex LH-20. Nuclear magnetic resonance (NMR) data were obtained using Bruker spectrometers (400 and 500 MHz). Chemical shifts are expressed in parts per million (ppm). The following chemicals were obtained from Sigma-Aldrich (Steinheim, Germany): α-glucosidase from *Saccharomyces cervisiae* (EC 3.2.1.20), *p*-nitrophenyl-α-D-glucopyranoside, acarbose, acetylthiocholine iodide (ATCI), galanthamine, 5,5-dithiobis-2-nitrobenzoic acid (DTNB), AChE enzyme (isolated from electric eels) (type VI-S lyophilized powder) and cyclooxygenase enzymes (0.3 µg protein, isolated from sheep seminal vesicle microsomes).

### 3.2. Plant Material-Collection and Authentication

The leaves of *C. menyharthii* were collected in May 2012 near Izindophi (on the road from Kranskop to the Thukela River, Umzinyathi District Municipality, KwaZulu-Natal, GPS lat 28.912814; long 31.008951 Alt 293 metres). The plant was identified by Mrs A. Young (Horticulturist, UKZN). A voucher specimen (Aderogba MA 03) was deposited at the Bews Herbarium (NU) of the University of KwaZulu-Natal. The collected plant material was oven dried at 45 °C for 3 days and then ground to powder.

#### 3.2.1. Extraction

The powdered plant material (1 kg) was extracted with 8 L of 20% aqueous methanol at room temperature for 24 h and filtered. The crude extract was concentrated *in vacuo* at 40 °C to about ⅓ of the filtrate original volume. This afforded the *C. menyharthii* crude extract (700 mL).

#### 3.2.2. Solvent Partitioning of the Crude Extracts

Concentrated crude extract (700 mL) was in turn sequentially extracted with *n*-hexane (3 × 800 mL), dichloromethane (3 × 800 mL), ethyl acetate (3 × 1 L) and finally n-butanol (700 mL). The solvent fractions were concentrated to dryness *in vacuo* to afford four solvent extracts: hexane (Hex, 4.0 g), dichloromethane (DCM, 5.2 g), ethyl acetate (EtOAc, 4.3 g) and butanol (6.5 g) fractions. 

### 3.3. Isolation of Compounds from *C. menyharthii* EtOAc Extract

The EtOAc fraction (4.0 g) was subjected to open silica gel column chromatography fractionation eluting first with hexane followed by an increasing gradient of ethyl acetate up to 100%, followed in turn with an increasing gradient of methanol up to 30%. The test tube fractions collected were analyzed on TLC plates using DCM/MeOH (9:1 and 4:1) as solvent systems. This afforded five fractions (A1-A5). Fraction A5 (750 mg) was further fractionated on Sephadex LH- 20 using DCM/MeOH (4:1) followed by an increasing gradient of methanol up to 35%. The test tube fractions collected were analyzed on TLC plate using DCM/MeOH (4:1) as solvent system. This afforded compound **1** (20 mg). Fraction A4 (450 mg) was fractionated on Sephadex LH-20 using DCM/MeOH (4:1) as eluent. The test tube fractions collected were analyzed on TLC plate using DCM/MeOH (4:1) as solvent system. This gave compound **2** (90 mg). Fraction A2 (60 mg) was subsequently purified on Sephadex LH-20 column using DCM/MeOH (9:1 and 4:1) as solvent mixtures. Analysis of the fractions collected on TLC plate using DCM/MeOH (4:1) as solvent mixture yielded compound **3** (3 mg). Purification of fraction A3 (300 mg) using Sephadex LH-20 column and DCM/MeOH (4:1) as solvent mixture afforded compound **4** (6 mg) on TLC analysis using DCM/MeOH (8.5:1.5) as solvent system.

### 3.4. Structure Elucidation of the Compounds

Structure elucidation of the isolated compounds was carried out using NMR (1D and 2D) spectroscopic techniques. The compounds were identified as:

*Myricetrin-3-O-rhamnoside (myricetrin)* (**1**): ^1^H-NMR (CH_3_OD, 500 MHz): Aglycone, δ: 6.95 (1H, s, H-2′), 6.95 (1H, s, H-6′), 6.36 (1H, d, J = 2.1 Hz, H-8), 6.20 (1H, d, J = 2.2 Hz, H-6), rhamnosyl Hs, δ: 5.32(1H, d, J = 1.5 Hz, H-1″), 4.23 (1H,dd, J = 3.3, 1.7 Hz, H-2″), 3.81 (1H, dd, J = 9.5, 3.4 Hz, H-3″), 3.37 (1H, t, J = 9.5 Hz, H-4″), 3.53 (1H, dd, J = 9.6, 6.2 Hz, H-5″), 0.97 (3H, d, J = 6.3 Hz, H-6″). ^13^C-NMR (CH_3_OD, 125.0 MHz): Aglycone, δ: 159.5 (C-2), 136.4 (C-3), 179.8(C-4), 163.3 (C-5), 99.9 (CH, C-6), 165.9 (C-7), 94.8 (CH, C-8), 158.5 (C-9), 106.0 (C-10), 122.0 (C-1′), 109.7 (CH, C-2′), 146.9 (C-3′), 138.0 (C-4′), 146.9 (C-5′),109.7 (CH, C-6′), rhamnosyl Cs, δ 103.7(CH, C-1″), 72.0 (CH, C-2″), 72.3 (CH, C-3″),73.4 (CH, C-4″), 72.1 (CH, C-5″), 17.7 (CH_3_, C-6″). The spectral data (^1^H and ^13^C-NMR) of compound **1** closely matched that of myricetrin-3-O-rhamnoside reported in the literature [16].

*Quercetin-3-O-rhamnoside* (**2**):^1^H-NMR (CH_3_OD, 500 MHz): Aglycone, δ: 7.34 (1H, d, J = 2.1 Hz, H-2′), 7.31 (1H, dd, J = 8.3, 2.1 Hz, H-6′), 6.92 (1H, d, J = 8.3 Hz, H-5′), 6.36 (1H, d, J = 2.2 Hz, H-8), 6.20 (1H, d, J = 2.1 Hz, H-8), rhamnosyl Hs, δ: 5.36 (1H, d, J = 1.6 Hz, H-1″), 4.23 (1H,dd, J = 3.3, 1.7 Hz, H-2″), 3.77 (1H, dd, J = 9.4, 3.4 Hz, H-3″), 3.36 (1H, t, J = 9.5 Hz, H-4″), 3.44 (1H, dd, J = 9.6, 6.1 Hz, H-5″), 0.95 (3H, d, J = 6.2 Hz, H-6″). ^13^C-NMR (CH_3_OD, 125.0 MHz): Aglycone, δ: 158.6 (C-2), 136.3 (C-3), 179.7 (C-4), 163.3 (C-5), 99.9 (CH, C-6), 165.9 (C-7), 94.8 (CH, C-8), 159.4 (C-9), 106.0 (C-10), 123.1 (C-1′), 117.1 (CH, C-2′), 149.9 (C-3′), 146.5 (C-4′), 116.5 (CH, C-5′),122.0 (CH, C-6′), rhamnosyl Cs, δ: 103.6 (CH, C-1″), 72.0 (CH, C-2″), 72.3 (CH, C-3″),73.4 (CH, C-4″), 72.1 (CH, C-5″), 17.7 (CH_3_, C-6″). The spectral data are in good agreement with the literature data [16].

*Quercetin* (**3**): ^1^H-NMR (acetone-d_6_, 400 MHz): δ: 12.2 (5-OH), 7.80 (1H, d, J = 2.1 Hz, H-2′), 7.70 (1H, dd, J = 8.5, 2.1 Hz, H-6′), 7.00 (1H, d, J = 8.5 Hz, H-5′), 6.51 (1H, d, J = 2.3 Hz, H-8), 6.26 (1H, d, J = 2.3 Hz, H-8), ^13^C-NMR (acetone-d_6_, 125.0 MHz): δ: 146.0 (C-2), 136.8 (C-3), 176.7 (C-4), 162.3 (C-5), 99.3 (CH, C-6), 165.3 (C-7), 94.5 (CH, C-8), 157.8 (C-9), 104.1 (C-10), 123.8 (C-1′), 115.8 (CH, C-2′), 147.1 (C-3′), 148.5 (C-4′), 116.2 (CH, C-5′),121.5 (CH, C-6′), the spectral data of compound **3** closely matched that of 3,3', 4',5,7-pentahydroxyflavone (quercetin) reported in the literature [17]. Quercetin is a flavonoid that is widely distributed in the plant kingdom.

(*E)-N-(4-hydroxycinnamoyl)-5-hydroxytryptamine, [trans-N-(p-coumaroyl) serotonin]* (**4**). ^1^H-NMR (acetone-d_6_, 400 MHz), δ: 7.45 (1H, d J = 15.7 Hz, H-7′), 7.40 (1H, d, J = 8.6, Hz, H-2′), 7.40 (1H, d, *J* = 8.6 Hz, H-6′), 7.16 (1H, d, J = 8.6 Hz, H-7), 7.03 (1H, s, H-1), 6.97 (1H, d, J = 2.2 Hz, H-4), 6.79 (1H, d, J = 8.6 Hz, H-3′),6.79 (1H, d, J = 8.6 Hz, H-5′), 6.67 (1H, dd, J = 8.6, 2.3 Hz, H-6), 6.40 (1H, d, J = 15.7 Hz, H-8′), 3.47 (2H, t, J = 7.2 Hz, H-9), 2.93 (2H, t, J = 7.2 Hz, H-10). ^13^C-NMR (acetone-d_6_, 125.0 MHz), δ: 124.0(CH, C-1), 112.6 (C-2), 129.4 (C-3), 103.6 (CH, C-4), 151.6 (C-5), 112.4 (CH, C-6), 112.5 (CH, C-7), 132.6 (C-8), 40.8 (CH_2_, C-9), 26.6 (CH_2_, C-10), 127.8 (C-1′), 130.2 (CH, C-2′), 116.6 (CH, C-3′), 159.9 (C-4′), 116.6 (CH, C-5′),130.2 (CH, C-6′),140.2 (CH, C-7′), 119.8 (CH, C-8′), 166.8 (C=O, C-9′), the spectral data (^1^H and ^13^C-NMR) of compound **4** closely matched that of (E)-*N*-(4-hydroxycinnamoyl)-5-hydroxytryptamine isolated from *Echinochloa utilis* reported in the literature [18].

### 3.5. Antimicrobial Bioassays

#### 3.5.1. Antibacterial Microdilution Bioassay

Minimum inhibitory concentration (MIC) values for antibacterial activity of the crude methanol extract, fractions and isolated compounds were determined using the microdilution bioassay in a 96-well (Greiner Bio-one GmbH, Frickenhausen, Germany) microtitre plates [19]. One hundred microlitres of the resuspended (in 70% aqueous acetone) crude extract, fractions (50 mg/mL) or compounds (1 mg/mL) was two-fold serially diluted with sterile distilled water, in duplicate down the microtitre plate for each of the four bacteria used. A similar two-fold serial dilution of neomycin (Sigma) (0.1 mg/mL) was used as a positive control against each bacterium. Water and 70% aqueous acetone were included as a negative/solvent controls. The screening was done in triplicate and repeated twice for each extract. Four bacterial strains were used; two Gram-positive (*Bacillus subtilis* ATCC 6051 and *Staphylococcus aureus* ATCC 12600) and two Gram-negative (*Escherichia coli* ATCC 11775 and *Klebsiella pneumoniae* ATCC 13883).

#### 3.5.2. Antifungal Microdilution Bioassay

The antifungal activity of the crude methanol extract, fractions and isolated compounds were evaluated against *Candida albicans* (ATCC 10231) using the micro-dilution assay [19], modified for an antifungal assay [20]. An overnight fungal culture was prepared in 10 mL yeast malt (YM) broth. Four hundred microliters of the overnight *Candida* culture were added to 4 mL of sterile saline solution. The absorbance was read at 530 nm and adjusted with sterile saline solution to match that of a 0.5 M McFarland standard solution. From this prepared stock, a 1:1000 dilution with sterile YM broth was prepared to give an approximately 10^6^ cfu/mL culture. One hundred microlitres of the resuspended (in 70% aqueous acetone) crude extract, fractions (50 mg/mL) or compounds (1 mg/mL) were two-fold serially diluted with sterile distilled water, in duplicate down a 96-well microtitre plate. A similar 2-fold serial dilution of amphotericin B (Sigma, 2.5 mg/mL) was used as a positive control. Water, 70% aqueous acetone and fungal free broth were included as negative/solvent controls. The screening was done in triplicate and repeated twice for each extract, the MIC and MFC values were recorded.

### 3.6. Enzyme Inhibition Bioassays

#### 3.6.1. Cyclooxygenase (COX-2) Inhibitory Bioassay

The COX-2 bioassays were performed as previously described [21]. Three units of human recombinant COX-2 enzyme containing a six histidine sequence near the N-terminus isolated from a Baculovirus overexpression system in S*f* 21 cells was used (Sigma-Aldrich). Sample volumes of 2.5 µL (10 mg/mL for the crude extract and fractions, and 1 mg/mL for the isolated compounds) were diluted in 17.5 µL of distilled water, giving a final assay concentration of 250 µg/mL for the crude extract and fractions, and 25 µg/mL for isolated compounds. The enzymes were activated with 1250 µL of co-factor solution (0.6 mg/mL L-adrenaline and 0.3 mg/mL reduced glutathione in 0.1 M Tris buffer, at pH 8.0) and pre-incubated on ice for 5 min. In 1.5 mL Eppendorf tubes, 60 µL of the enzyme solutions were added to each sample solution in duplicate and the mixture was incubated at room temperature for 5 min. Two separate sets of Eppendorf tubes, labelled the background (in which the enzyme was inactivated with 10 µL of 2N HCl before incubation), solvent blank (containing water instead of sample) and positive control (containing 100 µg/mL indomethacin obtained from Sigma) were included in the test. After 5 min of incubation at room temperature, the reaction was started by adding 20 µL ^14^C-arachidonic acid (16 Ci/mol, 30 µM) to each Eppendorf tube. The preparations were incubated in a water bath at 37 °C for 10 min and afterwards the reaction was stopped by adding 10 µL of 2N HCl except in the background tubes. The percentage activities were calculated using Graph Pad Prism (version 5.0) statistical software programme for Windows (GraphPad Software Inc.) and are presented as means ± standard errors of two independent experiments, each experiment in duplicate.

#### 3.6.2. Acetylcholinesterase (AChE) Inhibitory Bioassay

Inhibition of AChE by the crude methanol extract, fractions and isolated compounds was done as described by Ellman *et al.* [22] with some modifications. The acetylcholinesterase enzyme activity was measured by spectrophotometric observation of the increase in a yellow colour produced from thiocholine when it reacts with the dithiobisnitrobenzoate ion. AChE (isolated from electric eels) (type VI-S lyophilized powder) was obtained from Sigma-Aldrich. The assay was carried out in a 96-well microtitre plate. The crude methanol extract, fractions and isolated compounds were at initial concentrations of 10 mg/mL (crude extract and fractions) and 1 mg/mL (pure compounds). The increase in absorbance due to the spontaneous hydrolysis of the substrate was corrected by subtracting the ratio of reaction before adding the enzyme from the rate after adding the enzyme (0.2 U/mL). Percentage of inhibition was calculated by comparing the reaction rates for the sample to the negative control. Results were presented as means ± standard errors of the experiment in duplicate. The IC_50_ values of extracts and compounds were calculated using Graph Pad Prism (version 5.0) statistical software programme for Windows (GraphPad Software Inc.).

#### 3.6.3. α-Glucosidase Inhibitory Activity

α-Glucosidase inhibitory activity was determined as previously described by Tao *et al*. [23] with modifications as detailed by Rengasamy *et al*. [24]. Briefly, yeast α-glucosidase (0.1 Unit/mL) was dissolved in 0.1 M potassium phosphate buffer (pH 6.8), this was used as the enzyme solution. The control experiment contained the same reaction mixture, but the sample solution was replaced with the same volume of phosphate buffer. Acarbose dissolved in dimethyl sulphoxide (DMSO), was used as a positive control. The determinations were carried out in triplicate. The percentage inhibition (%) was calculated using the following equation:

% Inhibition = (A_control_ − A_sample_)/A_control_ × 100 (1)

where A_control_ is the absorbance of the control and A_sample_ is the absorbance of the sample. The IC_50_, which is the concentration of the sample required to inhibit 50% of the enzyme was determined for each sample.

### 3.7. Statistical Analysis

The statistical analysis to compare the means of the percentage inhibitions and the IC_50_ values was performed using SPSS^®^, version 21.0 for Windows (IBM, Chicago, IL, USA). Data on percentages and IC_50_ were arcsine transformed before being subjected to one-way analysis of variance (ANOVA) for Windows. Where there were significant differences (*p* ≤ 0.05), the means were further separated using Duncan’s multiple range test (DMRT) and/or Least Significant Difference (LSD).

## 4. Conclusions

Investigation of *Croton menyharthii* ethyl acetate soluble fraction of crude leaf extracts for its bioactive constituents afforded three flavonols and an indole alkaloid. These isolated compounds are reported for the first time from this plant species. The crude extracts, fractions and isolated compounds exhibited a broad spectrum of activities in antimicrobial and enzyme inhibition assays. This warrants the ethnomedicinal use of the plant in folk medicine.
